# Versatile Adjustment of LDPE Properties via Specific Treatments to Design Optical Components for Display Technologies

**DOI:** 10.3390/polym17050578

**Published:** 2025-02-22

**Authors:** Andreea Irina Barzic, Iuliana Stoica, Mihaela Iuliana Avadanei, Raluca Marinica Albu, Dan-Gheorghe Dimitriu, Dana-Ortansa Dorohoi

**Affiliations:** 1 Department of Physical Chemistry of Polymers, “Petru Poni” Institute of Macromolecular Chemistry, 41A Grigore Ghica Voda Alley, 700487 Iasi, Romania; irina_cosutchi@yahoo.com (A.I.B.); stoica_iuliana@icmpp.ro (I.S.); mavadanei@icmpp.ro (M.I.A.); 2 Faculty of Physics, “Alexandru Ioan Cuza” University, 11 Carol I Blvd., 700506 Iasi, Romania; dimitriu@uaic.ro

**Keywords:** polyethylene, refractivity, morphology, optical retarders, optical filters

## Abstract

Transparent polymers are key materials for producing a broad category of optical components. For specific uses, the material needs additional adaptation of its basic properties. In this context, the current article is focused on applying two kinds of treatments for tailoring the optical and morphological features of low-density polyethylene to match the criteria as optical retardation plates or band-pass filters. The first kind of treatment involves combined mechanical stretching (at various degrees) and abrasion. The second type of treatment resides in polymer foil exposure to plasma and immersion in a solution of a triphenylmethane derivative. For optical compensation purposes, the polymer foils were subjected to combined mechanical treatments consisting of stretching (at various degrees) and abrasion. To assess the level of produced molecular ordering, the polyethylene films were subjected to polarized infrared spectral measurements, polarized refractometry tests and morphological analyses by polarized light microscopy and atomic force microscopy. The results indicated that inducing birefringence and morphology anisotropy of samples leads to proper optical retardation. For optical filter purposes, the dyed polymer was shown to have changes in colorimetric parameters and morphological features and absorbed radiation in the interval of 480–660 nm, while others were transmitted. These characteristics are adequate for band-pass filter uses.

## 1. Introduction

Polymers represent an endless source of materials that are currently spread in almost all industries and are found in numerous products from our daily life. Regardless of their natural or synthetic origin, most of the macromolecular compounds have several advantages, such as a light weight, facile processing into various shapes, mechanical resistance, electrical insulation and optical clarity in the visible range [1,2]. Among the aforementioned features, transparency is a paramount characteristic for optical uses and describes the material’s ability to enable the propagation of optical radiation through it [3]. This optical property is what differentiates transparent plastics from opaque ones and recommends them for specific applications, like band-pass filters [4], shielding layers for photovoltaics [5], encapsulants for light-emitting diodes [6], optical lenses [7] and flexible photodetectors [8]. There is a variety of polymer structures that are used in these applications due to their elevated transparency, such as polyethylenimine, cellulose ethers, poly(methyl methacrylate) (PMMA), polyvinyl alcohol (PVA), alicyclic polyimides and so on [4,5,6,7,8]. Further developments can be attained by fine-tuning both the transmission (*T*) and refractive index (*n*) [9]. As a result, the use of such materials in optical applications that demand high transparency can grow. Additionally, if a transparent polymer foil gains molecular ordering, its optical behavior becomes anisotropic and interesting phenomena, like double refraction, take place [10]. Many polymer materials display an amorphous structure which displays isotropic optical properties. Therefore, to acquire molecular alignment, the action of external factors is needed. The literature reveals that the exposure of polymers to physical factors, like electric fields [11], acoustic waves [12], coherent light beams [13] and mechanical forces (shearing [14], stretching [15,16], abrasion [17] and combinations of these [18,19]) determines the augmentation of the optical anisotropy, hence producing a stronger birefringence (*Δn*) of the material. Other approaches, such as polymer chemical modifications [20] or polymer dispersed liquid crystals [21], are more complex and time consuming, while the level of generated optical anisotropy is harder to be matched to applicative purposes. This is why chemical methods are combined with physical ones to produce the desired molecular ordering [22]. The latter can be quantified by the magnitude of the difference between the light propagation speeds along the deformation direction and orthogonal to it. This aspect is of particular importance when designing optical retardation films (ORFs) [23]. Such optical components are often employed in display technologies (liquid crystal displays (LCDs), organic light-emitting diode displays (OLEDs)) for improving the viewing angle [24] and the wavelength dispersion dependence [25]. The optical performance of displays with incorporated ORFs depends on the optimization of the in-plane retardation (*Re*) and the out-of-plane retardation (*Rth*) [23]. Since these parameters are affected by the magnitude of the sample thickness and birefringence in various planes, it is essential to study the in-plane birefringence and out-of-plane birefringence for the accurate description of the optical anisotropy of materials used as ORFs in displays. Additionally, the image quality (color and contrast) on screens is related to the Δ*n* dispersion of the ORFs [26].

On the other hand, the *Δn* is linked to the response of the polymer chemical structure to external orientation forces. Among the manufacturing processes of ORFs, mechanical deformation is the most utilized. Several types of polymers were subjected to such orientation procedures to make ORFs, including PMMA [27], polystyrene [27], cellulose triacetate [28], polyimide [29], cellulose acetate [30], PVA [31], polycarbonate [32], polyethylene terephthalate [33,34] and polyethylene [35]. The latter polymer is a synthetic resin, characterized by lightweight, thermoplasticity, chemical stability, high impact strength, low moisture absorption, good transparency and large flexibility [36]. Comparatively to common polymers used for ORFs, it is important to highlight the advantageous stretchability (higher elongation at break) of polyethylene [37] in regard to polycarbonate [38] or PMMA [39], which enables better control of the molecular ordering and implicitly on the birefringence. Given these features, there are several studies on mechanically-induced birefringence in polyethylene [35,40,41,42,43]. Literature findings show that stretching is responsible for orientation of both amorphous and crystalline zones of polyethylene [40]; Δ*n* distribution is influenced by temperature and percentage in elongation, while the measuring wavelength [41,43] and exposure to γ-radiation [42] are other factors affecting the magnitude of the Δ*n*. Among these articles, there are only two reports [35,43] that link the Δ*n* property of polyethylene to ORF applications. However, the study of Emam-Ismail [35] does not clearly specify the method of inducing molecular ordering and does not directly measure the Δ*n* of this polymer, and instead calculates Δ*n* from the channeled spectra method. The study of Nagib et al. [43] deals with spectral dependences of the retardance and Δ*n*, where the first one is determined based on their proposed calibration model, emphasizing the impact of foil thickness on the birefringent behavior of the plate under variable manufacturing conditions.

Another important optical component of current display technologies is the optical filters. Particularly, band-pass filters (BPFs) enable the attenuation of a specific wavelength zone and transmit outside the remaining spectral domain. Some of these filters operate based on absorption phenomena and they are obtained by the incorporation of specific dyes in transparent polymers [4]. The key properties of BPFs are the steep slopes (suitable isolation of targeted frequencies) and powerful blocking (diminishment of unwanted radiation leakage) [44]. A relatively recent work showed that BPFs are important components that can enhance the rendered colors of a LCD [45].

Our previous reports were focused on investigating the relationships between the molecular ordering induced by combined mechanical treatments (e.g., abrasion and corrugated surface pressing [18], shearing and stretching [19] and shearing, drawing and scratching [31]) and the resulted birefringence measured by various methods (e.g., polarizing ellipse, channeled spectra, interferometry, compensatory method and polarized light refractometry) [46]. Moreover, optical birefringence can be correlated with chemical structure by infrared dichroism measurements [40], but also techniques like X-ray diffraction [40], polarized light microscopy [47] and atomic force microscopy [48] can evidence the molecular ordering induced in polymers. Another previous work [49] emphasized the role of solvent type on optical performance of BPFs made of polyvinyl alcohol mixed with various amounts of crystal violet (CV).

The present article is a continuation of previous works and currently explores the possibility to manage the birefringence magnitude via combined mechanical treatments (i.e., stretching and abrasion), which are applied for the first time to low-density polyethylene (LDPE) foils. Novel insights are found from examination of the molecular structure ordering by infrared dichroism and also from deep investigation of the optical and morphological anisotropy by polarized refractometry and microscopy techniques. These results are paramount for carful design of polymers as ORFs for display technologies. Additionally, a treatment of plasma exposure followed by dye immersion of an LDPE film is done for fabrication of BPFs, and to our knowledge such aspects are not discussed in the literature yet. The results of this study prove that the prepared material is suitable for blocking optical radiation in the 480–660 nm range, whereas the others are transmitted—as needed for the pursued applications.

## 2. Materials and Methods

### 2.1. Sample Preparation

The polymer used in this work is low-density polyethylene (LDPE) in the form of transparent foil. LDPE films having length of 6.4 cm and width of 1.1 cm were subjected to the first category of treatment, which consisted of combined mechanical deformations in two steps. The first step involved the mechanical stretching of the LDPE foil with a lab-made device, which was constructed based on the following elements: a static part of metallic plates that immobilizes one end of the sample and a mobile part of metallic plates that hold the opposite end of the sample and facilitate uniaxial elongation via the assembly, driven by turning the knob of the perpendicular set screw. The stretching was done along the sample length and under mild temperature (45 °C) conditions. The elongated foils were quickly cooled with cold air for preventing the relaxation phenomenon. Here, four stretching levels were attained (see Table 1) and all drawn foils were used for the second step of abrasion treatment. Sample rubbing was done on a lab-designed device, along the stretching direction, in identical conditions (regardless of the drawing level). The abrasion device mainly consists of a parallelepiped covered on the bottom side with sandpaper (120 grit). The parallelepiped part wrapped with abrasion material was kept in close contact with sample surface (invariable abrasion pressure being ensured by pressing a 1 kg weight) and moved with fixed speed of 5 mm/s, thus scratching the fixed polymer foil.

The names of the drawn and rubbed samples were chosen as follows: LDPE 0 (no stretching and no abrasion), LDPE 1 (first level of stretching + abrasion), LDPE 2 (second level of stretching + abrasion), LDPE 3 (third level of stretching + abrasion) and LDPE 4 (fourth level of stretching + abrasion). Table 1 summarizes the variations of the dimensions (length and width) corresponding to initial and mechanically treated LDPE films and also the ratio between the final (after drawing) and original length, noted *Rs*. Table 1 additionally lists the sample thickness, which was determined with digital micrometer model 1108-150 (Insize Co. Ltd., Suzhou New District, Suzhou,China). The samples were replicated three times.

The second category of treatment implicated the functionalization of both sides of the LDPE 0 film by exposure for 3 min in diffuse coplanar surface barrier discharge (DCSBD) plasma using RPS40+ device (Roplass s.r.o., Modřice, Czech Republic). Afterwards, the polymer layer was immersed in a 1% ethanol solution of triphenylmethane derivative (crystal violet (CV)) for 16 h. Then, the LDPE 0_CV sample was left to dry under dark conditions.

### 2.2. Characterization

Polarized light microscopy (PLM) was carried out on ADL 601P microscope (Bresser, Rhede, Germany) under crossed polarizers.

Atomic force microscopy measurements were performed on a NTEGRA system provided by NT-MDT Spectrum Instruments Company from Zelenograd, Moscow, Russia. A NSG 10 cantilever (TipsNano OÜ, Tallinn, Estonia) with a resonance frequency of 172 kHz and a normal spring constant of 10.7 N/m was utilized to investigate the surface texture of the samples in environmental circumstances at 21 °C. The tip–surface interaction type was semicontact. For image acquisition and morphology, furrows, functional and spatial analyses, Nova 1.1.1.19891 and Image Analysis 3.5.20102 (NT-MDT Spectrum Instruments, Zelenograd, Moscow, Russia) and MountainsSPIP^®^ Academic 10 (Digital Surf, Besançon, France) software was employed.

The polarized ATR-FTIR spectra were recorded by using a Bruker Vertex 70 spectrometer (Bruker Optics GMBh, Ettlingen, Germany) equipped with the Golden Gate^TM^ accessory and a wire grid polarizer from Perkin Elmer. The measurements were made for a complete rotation of the polarizer (0–360°), in steps of 15°, with 256 scans at 2 cm^−1^ resolution. Two experimental setups were used: machine direction (MD) and transverse direction (TD), by placing the sample on the ATR crystal with the uniaxial stretching direction aligned parallel and perpendicular, respectively, to the IR beam direction. The calculus of the dichroic ratios used the TE wave in the MD and TD experimental set-ups.

UV-VIS spectra were attained on a SPECORD 210 PLUS instrument (Analytik Jena GmbH, Jena, Germany).

Birefringence measurements were undertaken on a DR M4 device (Atago Co. Ltd., Saitama, Japan) which was equipped with a polarizer and four wavelength filters (486, 589, 656 and 670 nm).

Colorimetry tests were done on a CL-70F setup (Konika Minolta, Tokyo, Japan).

## 3. Results

This work investigates novel insights extracted from the correlation between the structural, morphological and optical features of LDPE films that were processed by specific approaches for designing either ORF or BPF components for display technologies.

### 3.1. Analyses of LDPE Films for ORF Applications

The LDPE foils were mechanically processed by combined stretching and rubbing for rendering optical and morphological anisotropy, which are needed for ORF uses.

#### 3.1.1. Dichroic ATR-FTIR Spectroscopy

**Figure 1:**
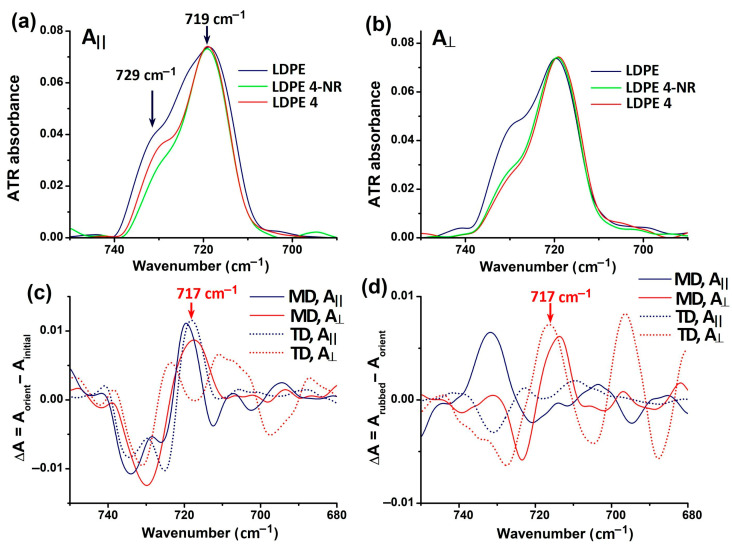
ATR-FTIR spectra in the spectral region of CH_2_ rocking vibrations of initial LDPE, LDPE 4-NR (just drawn) and LDPE 4 (drawn and rubbed), recorded in machine direction (MD) with light polarized (**a**) parallel and (**b**) perpendicular to the stretching direction; (**c**) difference spectra between LDPE 4-NR and initial LDPE 0, recorded in machine direction and transverse direction (MD and TD); (**d**) difference spectra between LDPE 4 and LDPE 4-NR.

Infrared polarization studies of polyethylene films have shown the dichroic character of the deformation vibrations of methylene groups in the main chain, reflecting the average orientation of the amorphous and crystalline domains [50,51,52]. Under uniaxial stretching, some of the *gauche* sequences are converted to *trans* and the orthorhombic unit cell might transition into a monoclinic one [51,52]. Therefore, the intensity and position of characteristic vibrations of methylene groups exhibit changes accordingly. In this work, we put emphasis on the in- and out-of-phase CH_2_ rocking vibrations peaking in the 750–700 cm^−1^ domain, whose polarization is directly linked to the direction of crystallographic axes. Figure 1a,b shows the γ_rock_(CH_2_) band recorded in parallel and perpendicular polarization, respectively, for the initial LDPE 0, LDPE 4-NR (non-rubbed) and LDPE 4.

Two close components are seen: the sub-band at 729 cm^−1^ corresponds to a pure crystalline vibration, characteristic to an orthorhombic unit cell, and is polarized along the *b*-crystallographic axis. The component around 719 cm^−1^ is polarized along the *a*-crystallographic axis and is a contribution from methylene groups in both amorphous and crystalline domains [50,51,52]. Generally, the unoriented polyethylene contains both bands, with the 719 cm^−1^ vibration being more intense than the crystalline band. The uniaxial stretching of LDPE of 400% led to the decrease of the crystalline band (green trace in Figure 1a,b) and a slight redshifting of the maximum. The first effect was visible when LDPE 4-NR was analyzed in the machine direction (MD) with IR radiation polarized parallel and perpendicular on the stretching direction. The second effect can be more clearly seen in the FTIR spectra recorded in the transverse direction with a perpendicularly polarized IR beam. The difference spectra [LDPE 4-NR–LDPE] in Figure 1c show a weak positive band around 717 cm^−1^, evidenced in parallel polarized light and that arises when uniaxial stressing the film. Siesler [51] correlated the band at 717 cm^−1^ with the monoclinic crystal phase of polyethylene. This means that the main conversion from orthorhombic to monoclinic, although in a low extent, took place in LDPE 4-NR in the elongation direction and the crystals are oriented in this direction as well. The effect of abrasion the LDPE 4-NR film, observed in Figure 1d, is captured with light polarized perpendicular on the stretching direction. This fact suggests that abrasion caused the development of a small fraction of monoclinic crystal phase in a direction perpendicular to the rubbing and the stretching direction. In the same time, a small recrystallization of the original orthorhombic order is seen when analyzed with parallel polarization (Figure 1c), indicating the abrasion simultaneously provoked a fraction of chains to return to the initial arrangement.

The effect of recrystallization has been evidenced for polyethylenes with a strain factor of more than 150% [51]. In this case, elongation causes unfolding of molecular chains and disruption of the orthorhombic cell, both of which leveled off between 50 and 150% strain. Beyond this value, Siesler [51] observed that increasing the stress caused only the recrystallization of unfolded chains and a better orientation of them.

By using the γ_rock_(CH_2_) vibrations recorded in polarized light, the distortion of the orthorhombic cell upon stretching and rubbing can be analyzed in terms of the structural absorbances, *A*_0_, of the two bands (Equation (1)) [51]. The $A_{0.729}/A_{0.719}$ ratio is a measure of the deformation degree of the unit cell, eliminating the thickness variation as a result of elongation. Further on, orientation of the *a*, *b* and *c* crystallographic axes are calculated by using the dichroic ratios of the two rocking vibrations (Equations (2)–(5)) [51]:

(1) $$A_{o}=\frac{A_{∥}+2A_{⊥}\;}{3},$$

(2) $$f_{a}=\frac{R_{729}-1}{R_{729}+2}\;,$$

(3) $$f_{b}=\frac{R_{719}-1}{R_{719}+2},$$

(4) $$R=\frac{A_{∥}}{A_{⊥}},$$

(5) $$f_{a}+f_{b}+f_{c}=0\;,$$

**Figure 2:**
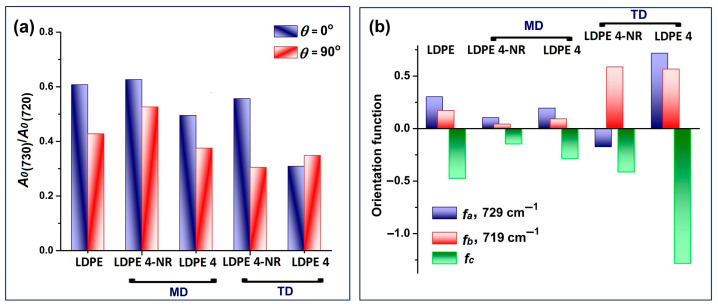
FTIR spectral analysis of the rubbing process, focused on γ_rock_(CH_2_) in crystalline domains (729 cm^−1^) and amorphous + crystalline domains (719 cm^−1^): (**a**) Structural absorbance $A_{0.729}/A_{0.719}$; (**b**) Calculated orientation functions of the crystallographic axes, *f_a_*, *f_b_* and *f_c_*.

The crystallographic axis *c* is the direction of the polyethylene chain. Variation of the structural factor $A_{0.729}/A_{0.719}$ induced by elongating the film, followed by abrasion, is presented in Figure 2a. The decrease of $A_{0.729}/A_{0.719}$, related to a decrease of the uniformity of the orthorhombic unit cell, is evident after rubbing the LDPE 4-NR film and is better captured in transverse direction.

The orientation functions of the crystallographic axes, *f_a_*, *f_b_* and *f_c_*, were used to analyze the behavior of lamellar units and chain unfolding for polyethylene samples [50,51]. In LDPE 4-NR and for the FTIR spectra recorded in the MD setup, all three parameters have lower values as compared to initial LDPE 0 film (Figure 2b), which would suggest unfolding of the chains and that the direction of stretching does not match any of the three axes. On the contrary, measurements in the TD setup show that stretching led to a significant increase of *f_b_* and relative decreases of *f_a_* and *f_c_*. In this case, the lamellar units orient as a whole with the *f_b_* axis perpendicular to the direction of drawing. The abrasion process further strengthened the lamellar orientation and chains alignment, with the *c* axis parallel to the direction of rubbing and of elongation.

**Figure 3:**
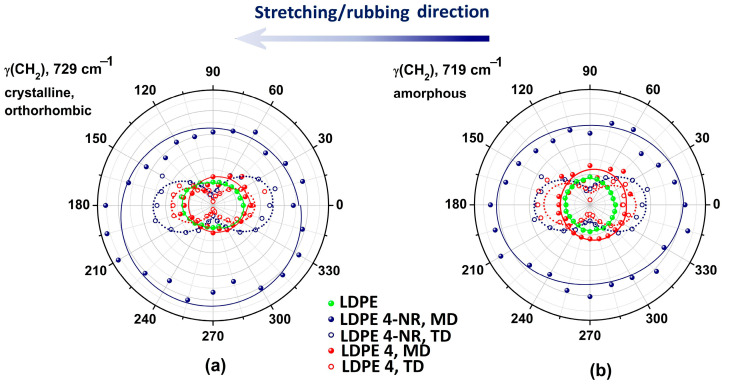
Anisotropy of *γ_rock_*(CH_2_) with the polarization angle of the IR beam, induced by the uniaxial stretching and rubbing, for the (**a**) crystalline band, at 729 cm^−1^, and (**b**) amorphous band, at 719 cm^−1^.

Orientation of the dipole moments of the two γ_rock_(CH_2_) vibrations in relation with the polarization angle of the IR beam is visualized in the polar diagrams in Figure 3. The initial centrosymmetric distribution of the crystalline phase in the LDPE film has a change of symmetry and became bilobate after abrasion, while the stretching led only to the increase of the magnitude (Figure 3a). The maximum of the oscillating dipoles is on the 0–180° line, which is the mechanical force’s direction. Most chains in the amorphous phase did have the director oriented along the stretching direction before rubbing and became highly aligned in LDPE 4.

**Figure 4:**
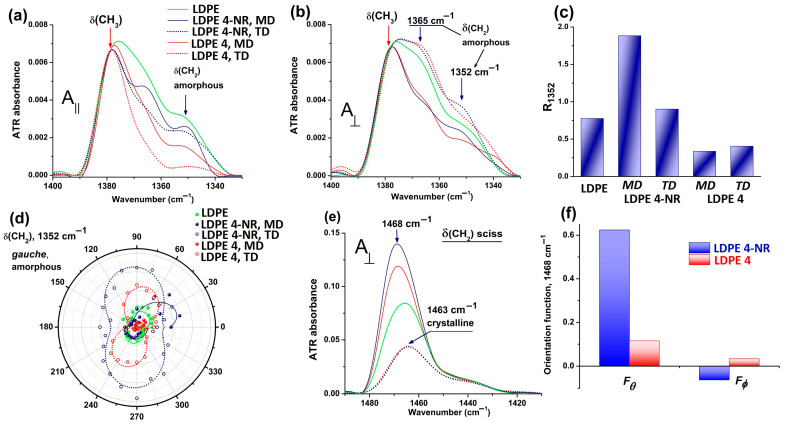
Polarized infrared analysis of the orientation of *gauche* conformers (bending vibration, 1352 cm^−1^) in amorphous phase of LDPE: (**a**) parallel polarization; (**b**) perpendicular polarization; (**c**) variation of the IR dichroic ratio; (**d**) anisotropy diagram as a function of the polarization angle, the experimental data (points) were fitted with a cos^2^*θ* function; (**e**) spectral variation of the scissoring vibration of methylene groups, used to determine the biaxial orientation functions (**f**).

Analysis of the 1500–1300 cm^−1^ spectral region provides another perspective of the LDPE 4-NR anisotropy before and after abrasion. Figure 4a,b presents the spectral changes induced by stretching (dark blue traces) and rubbing (red traces) on the LDPE film, recorded in parallel and perpendicular polarization. The bands at 1368 and 1352 cm^−1^ (deformation vibration) belong to methylene groups with a *gauche* conformation and that are located in amorphous phase, so they are linked to the amount of chain folds [53,54]. In parallel polarization (Figure 4a, the concentration of *gauche* conformers decreased after elongating the film and even more after abrasion. This decrease is best captured in the transverse direction (TD), and as well in perpendicular polarization (Figure 4b). The dichroic ratio of the *gauche* band at 1352 cm^−1^, *R*_1352_, increased after stretching the initial LDPE 0 film, (Figure 4c), but decreased further after rubbing. Therefore, a large number of chains in the amorphous phase become oriented in the stretching direction, but afterwards they align in a perpendicular direction after rubbing. This behavior is visible in Figure 4c through the value of *R*_1352_ in TD mode for LDPE 4. Orientation of the amorphous *gauche* conformers analyzed in the anisotropy diagram in Figure 4d is indeed almost perpendicular to the abrasion direction. The initial elliptic symmetric bending vibration changed after rubbing into a two-lobed symmetry, with a periodicity of 180°. However, stretching the initial LDPE 0 film produced an orientation of the *gauche* conformers in chain folds not in the stretching direction, but at a nominal angle of about 45–60°.

Analysis of the biaxial anisotropy of LDPE 4-NR and LDPE 4 was done by calculating two orientation functions that are related to the transition moment of the scissoring vibration of methylene groups (centered on 1468 cm^−1^, Figure 4e). The orientation of the transition dipole moment of *δ*(CH_2_)sciss can be decomposed in in two projections: in the film plane (*xy*) and out-of-plane (*z* plane). The corresponding components are orientation functions *F_θ_* and *F_ϕ_*, as follows [55]:

(6) $$F_{\theta }\left(xy\;plane\right)=\frac{A_{x}-\frac{A_{y}+A_{z}}{2}}{A_{x}+A_{y}+A_{z}}\;,$$

(7) $$F_{\phi }\left(z\;plane\right)=\frac{A_{y}-A_{z}}{A_{y}+A_{z}},$$

where *A_x_, A_y_* and *A_z_* are the absorbances of *δ*(CH_2_)sciss with the direction of the electric field of the IR beam polarized in the *x, y* and *z* directions. The *x* and *y* directions are in the plane of the LDPE film, while the value of *A_z_* is determined from the isotropic average absorbance $A_{0}=(A_{x}+A_{y}+A_{z})/3$. The angle *θ* is between the IR beam and the transition moment of *δ*(CH_2_)sciss, while *ϕ* is the angle between the *y* axis and the projection of the transition dipole moment on the (*x*, *y*) plane.

As shown in Figure 4f, the value of $F_{\phi }$ increased after rubbing, which could mean a rotation of the scissoring vibration in an out-of-plane direction or possibly, a fragment of polyethylene chain has acquired a tilted orientation in reference to the abrasion direction. The values of $F_{\theta }$, related to the in-plane component of the whole orientation function, are positive after stretching and becames negative after rubbing. It can be interpreted as the methylene groups are rather oriented in the plane of the film in the *x* direction after stretching and reorient themselves in an almost perpendicular direction after abrasion.

#### 3.1.2. Morphology Investigation

**Figure 5:**
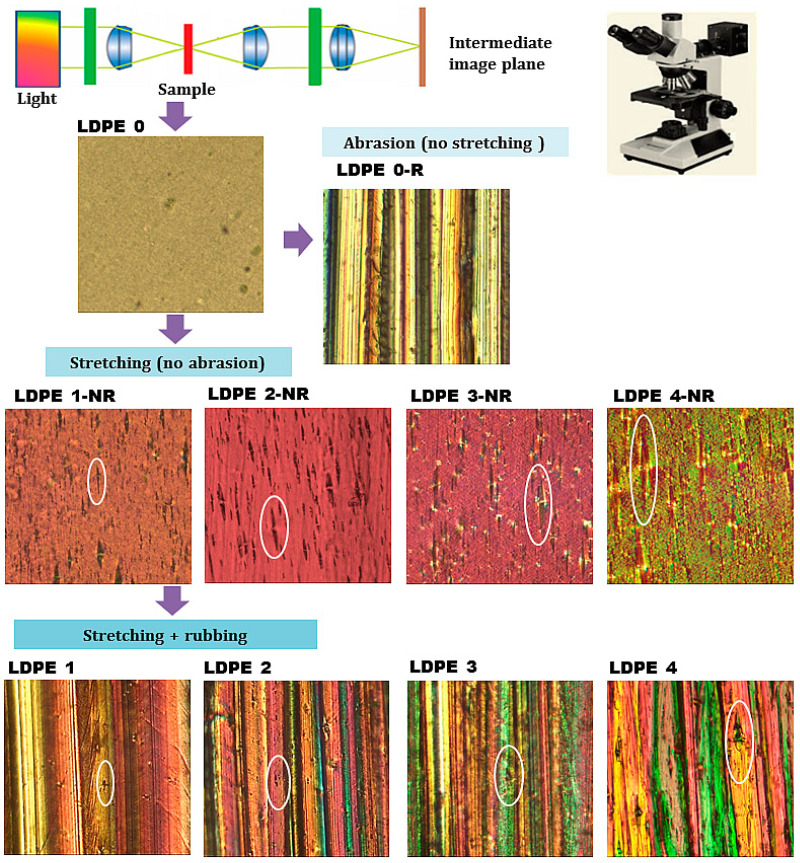
Polarized light microscopy images taken at magnification of 10× (under crossed polarizers) for all LDPE samples.

PLM is a suitable technique that helps to examine materials by emphasizing the changes in their refractive properties and colors. This method is especially useful when the analyzed specimen is characterized by molecular ordering, so quality and high-contrast images are achieved for birefringent layers. Based on these considerations, LDPE foils were inspected by PLM before and after the mechanical treatments and the recorded data are illustrated in Figure 5.

The micrograph corresponding to the pristine sample (LDPE 0) indicates that this film is characterized at micro-level by a relatively smooth surface with isotropic features. This observation is supported by other articles [56,57] that studied this polymer by optical microscopy and scanning electron microscopy methods. Further, Figure 5 contains PLM images of the polyethylene film subjected only to rubbing and no stretching (LDPE 0-R) and PLM images of the stretched polyethylene films in the absence of abrasion (LDPE 1-NR, LDPE 2-NR, LDPE 3-NR, and LDPE 4-NR). The sample treated by abrasion (LDPE 0-R) presents a morphology composed of parallel micro-trenches, which appear along the rubbing direction. This texture is formed due to the alignment of polymer chains in the superficial layers of the material generated by the rubbing force. The samples treated solely by uniaxial drawing (no abrasion) display a bulk orientation of the macromolecules, which in recorded micrographs are noted as the modification of initial spherulitic morphology into a fibrillar one; the latter being remarked as “stripes” disposed parallel to the stretching direction. These “stripes” have higher length as the drawing degree of LDPE is higher (see marked white oval zones in micrographs from Figure 5).

Upon application a two-step mechanical treatment of stretching and abrasion, the morphological aspects of the samples LDPE 1–LDPE 4 are modified, as seen in Figure 5. It is widely known that LDPE is an amorphous polymer (irregular arrangement of chains), but also contains regions of crystallinity where the macromolecules are organized and tightly packed [58]. During the uniaxial drawing of the LDPE, the macromolecules from both amorphous and crystalline areas start to adopt a preferential alignment along the direction of external forces of stretching and abrasion. As the stretching level increases, LDPE chains are gradually extended along the imposed orientation leading to a variation of the medium distance among the crystalline formations. The polymer layer is distorted along the deformation force so that the regularity of chain segments is gradually augmented by enhancing the drawing degree. This aspect is seen in the studied films as “stripes” formed parallel to deformation direction, which are increasing in length as the stretching level is increased from LDPE 1 to LDPE 4 (see marked zones in micrographs from Figure 5).

The literature [59,60] proves that at the macro-scale, the drawing process makes the original spherulitic morphology change into a fibrillar one parallel to the machine direction. Resembling features were also noted in the case of different semi-crystalline polymers [31,61]. Our previous works [31,62] demonstrated that the abrasion procedure with sandpaper acts on the superficial layers of the polymer and is responsible for appearance of parallel micro-trenches. Since abrasion of LDPE foils was applied in the same direction as stretching, leads to a supplementary molecular ordering, overlapping to that induced by drawing. This is why for the LDPE 1–LDPE 4 films some of the occurring “stripes” are remarked onto or in-between these trenches, as revealed in Figure 5. The proposed combination of mechanical treatments of the LDPE foils enables to reach a high level of chain orientation, which influences the anisotropy of morphology and also the optical anisotropy.

**Figure 6:**
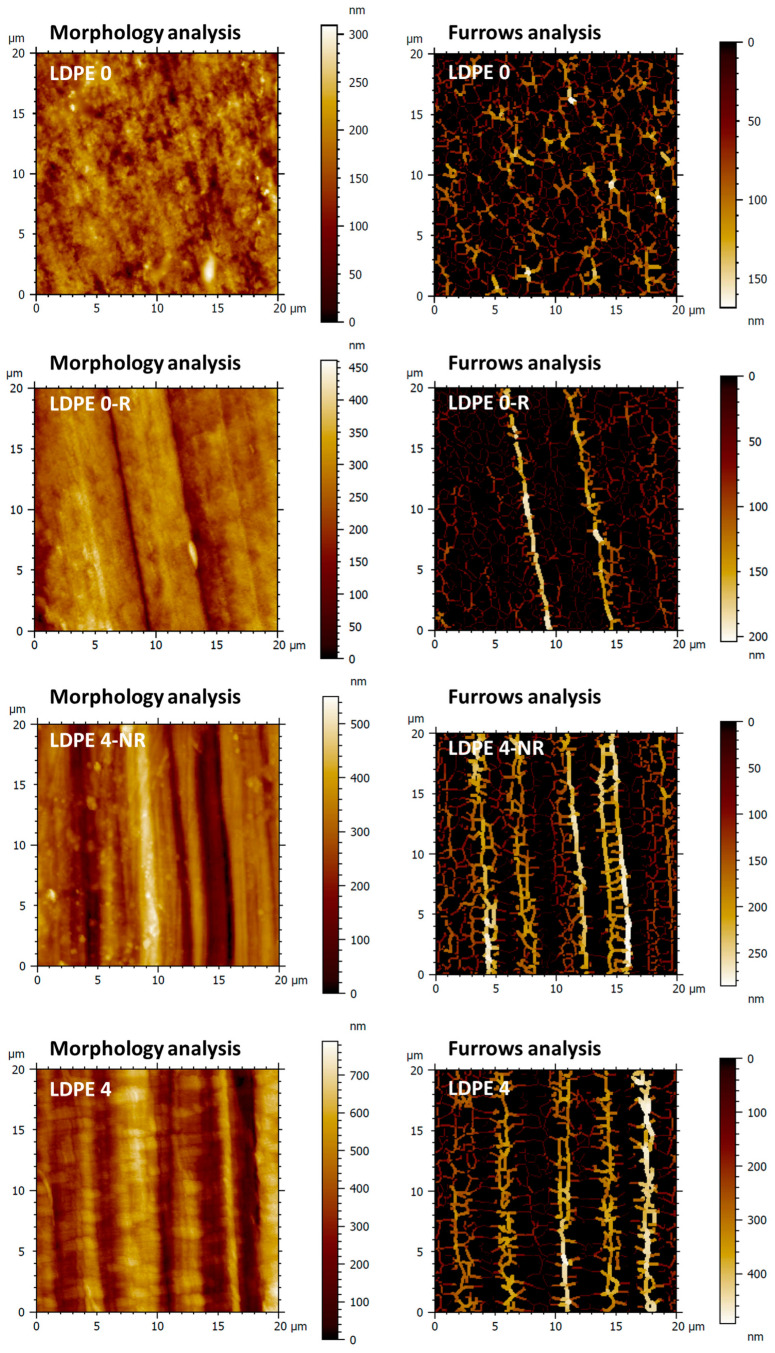
2D topographical images and corresponding furrow maps attained for pristine polymer film (LDPE 0), rubbed polymer film (LDPE 0-R), stretched polymer film (LDPE 4-NR) and double mechanically processed (stretched and rubbed) polymer film (LDPE 4) used in morphology and furrows analyses.

Deeper analysis is further undertaken at the nano-scale to emphasize how the chosen treatment affects the polymer surface features in relation to most efficient molecular ordering of the samples. Thus, AFM characterization was focused on the pristine film (LDPE 0), rubbed polymer film (LDPE 0-R) and stretched polymer film (LDPE 4-NR), with the highest stretching level and double mechanically processed (stretched at highest degree and rubbed) polymer film (LDPE 4). Figure 6 depicts the 2D topographical images obtained by AFM measurements.

The surface morphology analysis of the initial LDPE 0 film highlighted structural formations, distributed uniformly on the top surface that underwent significant visual alterations following the mechanical processing (LDPE 0-R, LDPE 4-NR and LDPE 4). Consequently, periodic micro-grooves were created on the surface, with their frequency varying according to the type of mechanical processing employed. While the rubbing process induced a periodicity of 34.84% and a period of 6 ± 0.6 μm and the stretching process a periodicity of 63.29% and a period of 4 ± 0.7 μm, by integrating these two processes, intermediate values for the periodicity and period (46.78% and 5 ± 0.5 μm, respectively) and a more evident and deeper structuring of the relief was achieved (LDPE 4). As a result, not only did the root mean square roughness (*Sq*) significantly increase by approximately 1.5 times for the rubbed polymer film, around 2.5 times for the stretched polymer film and about 3.5 times for double mechanically processed polymer film, but also the complexity of the morphological formations, described by the increase in the surface area ratio (*Sdr*) parameter (Table 2). According to [63], the surface roughness of a material mainly influences the transmittance and less the refractive index; hence, it has insignificant impact on the birefringence. Thus, AFM studies are further examining the surface topography in terms of the micro-valley networks and texture direction, which reveal information on molecular ordering and implicitly on birefringence.

In Figure 6, starting from the height AFM images, the furrows analysis described the micro-valley network through two different parameters, namely the maximum and the mean depth of furrows. The evolution of these characteristics (Table 2) signifies the emergence of well-differentiated valley–hill structures on the sample surface due to mechanical processing. The prevalence of valleys, progressively noticeable from one mechanical processing to another (LDPE 0-R < LDPE 4-NR < LDPE 4), was greater than initially observed in the unaltered sample (LDPE 0).

**Figure 7:**
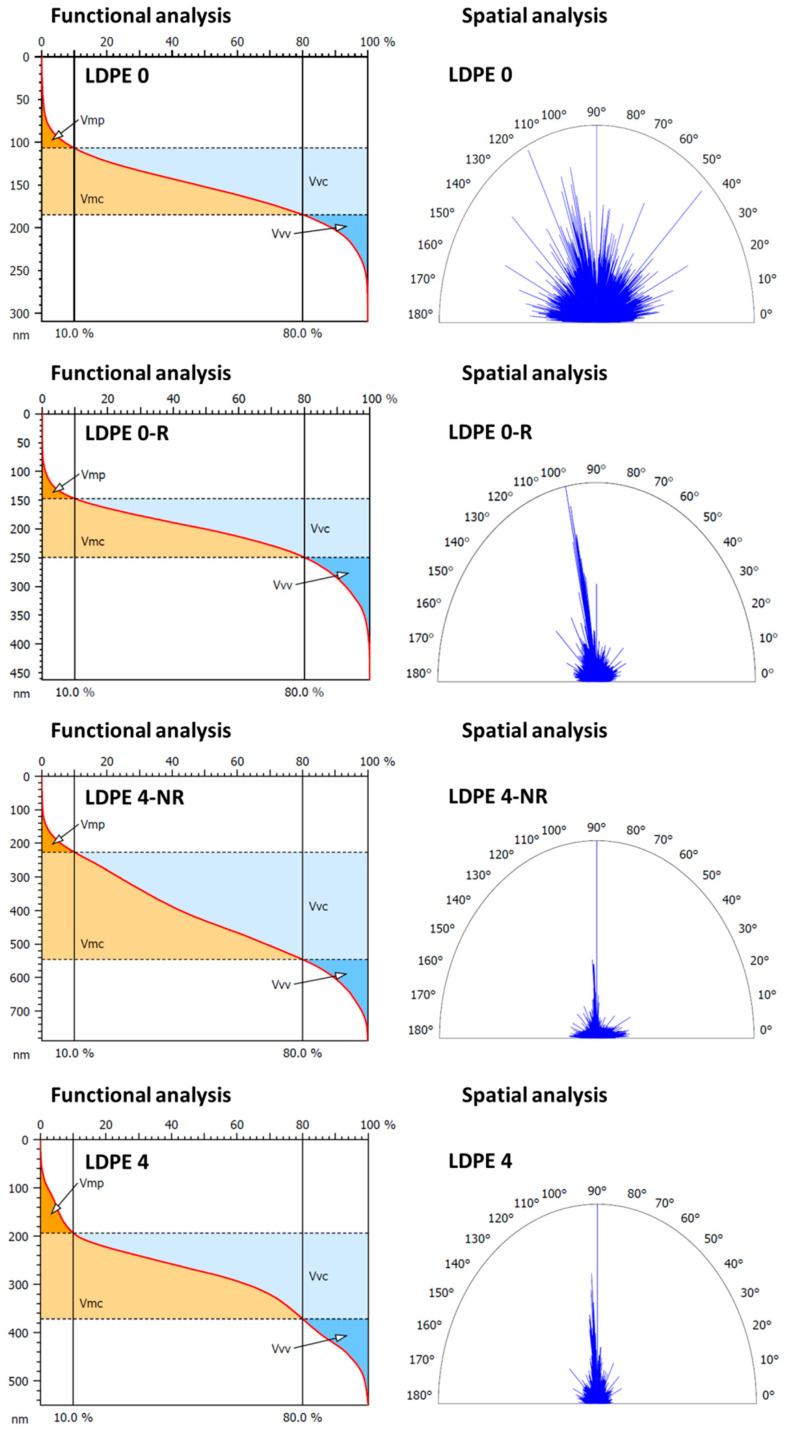
Abbott-Firestone curves and polar graphs of the texture direction obtained for pristine polymer film (LDPE 0), rubbed polymer film (LDPE 0-R), stretched polymer film (LDPE 4-NR) and double mechanically processed (stretched and rubbed) polymer film (LDPE 4) used in functional and spatial analyses.

This can also be emphasized through functional analysis (Figure 7), utilizing the parameters that delineate the evolution of the volume of remodeled material on the sample surface: peak material volume (*Vmp*) and core material volume (*Vmc*), alongside the volume of air that can be retained in the spaces within the central and lower regions of the relief: core void volume (*Vvc*) and valley void volume (*Vvv*) (Table 3).

By analyzing the Abott–Firestone curves from Figure 7 it can be observed that these functional volume parameters rise significantly after mechanical processing, indicating the formation of a more clearly defined relief, with a wider disparity between the levels of the valleys and the hills. The optimal results were achieved using twofold mechanically treated (stretched and rubbed) polymer film (LDPE 4), resulting in an increase of functional volume parameters by 2 to 4 times.

The spatial analysis realized by representation of the polar graphs of the texture direction (Figure 7) indicates for the pristine sample (LDPE 0) a texture direction index of the surface (*Stdi*) of 0.626, and thus a high degree of isotropy. Conversely, corroborating other studies, the mechanically processed samples exhibit a progressively significant enhancement in surface anisotropy, as evidenced by the low *Stdi* values (Table 3). This phenomenon arises from the emergence of long and uniform periodic morphological structures in the direction of mechanical processing. Despite the differences in morphological characteristics and textural parameters between LDPE 0-R and LDPE 4-NR samples, spatial analysis reveals that comparable values for the degree of anisotropy are achieved when the samples are processed independently, whether through abrasion or stretching (Table 3). This occurs despite the fact that the friction process predominantly influences the surface, while the stretching process impacts both the surface and the volume. By integrating the two mechanical processing methods, the enhancement of surface morphological anisotropy is significantly more pronounced, with *Stdi* dropping to 0.167 (Table 3).

All investigated 3D texture parameters suggest that, from a morphological perspective, LDPE 4 is an appropriate candidate for use as a retardation polymer film, as it enhances the birefringence and, consequently, the optical retardation by promoting a greater degree of induced molecular ordering in the polymer film by dual mechanical processing.

#### 3.1.3. Transparency and Birefringence Dispersion

**Figure 8:**
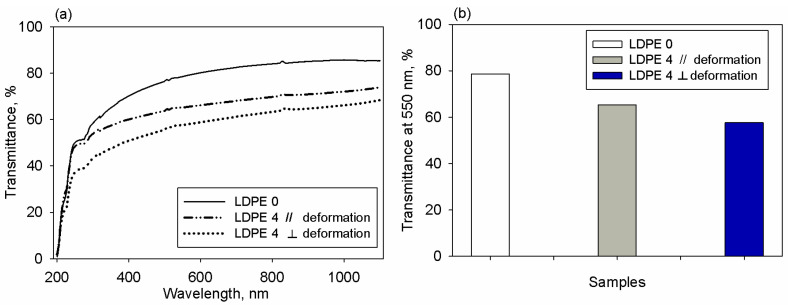
Transmittance versus wavelength for LDPE 0 and LDPE 4, measured parallel and orthogonal to deformation direction (**a**) and changes recorded in transmittance at 550 nm for the selected samples (**b**).

UV-VIS analysis of the pristine polymer foil LDPE 0 was undertaken to evaluate its level of transparency in a wide spectral domain of 200–1100 nm. As one may see in Figure 8a, this polymer displays a relatively high transmittance, starting with 450 nm, and its values increase towards higher wavelengths. For comparison reasons, UV-VIS spectra were also registered for the polymer foil with highest molecular ordering induced by drawing and treated by abrasion, namely LDPE 4. These spectra were collected for two cases, namely parallel and perpendicular to the direction of the deformation imposed to the sample (see Figure 8a). To get a clearer conclusion, the transmittance data measured at 550 nm were graphically plotted for LDPE 0 and LDPE 4 film (in parallel and orthogonal position), as observed in Figure 8b. The magnitude of the transmittance is highest for the polymer film lacking mechanical treatment (LDPE 0). Stretching and rubbing along the polymer film length determines the reduction of the transparency, as noticed in Figure 8b. This might occur because in the semi-crystalline polymers the refractive indices of the crystallites along the three directions are different (owing to mechanical induced anisotropy) even more than those of the amorphous phase [64,65]. Hence, visible radiation is scattered at the multiple boundaries between these crystalline and amorphous domains [64,65], and thus this phenomenon allows less light to be transmitted through the oriented polymer layer, as in the case of stretched and rubbed LDPE 4 foil. Moreover, there are differences in the transmittance values recorded when the sample is in a parallel or perpendicular position, as shown in Figure 8b. According to [66], a material absorbs a higher amount of optical radiation having the electric vector orthogonal to the imposed strain and rubbing direction comparatively to the case where light has the electric vector parallel to the deformation direction. This explains the anisotropy of the transmittance of the LDPE 4 film.

Another important optical parameter in ORF applications is the refractive index (*n*), which is linked to the speed of optical radiation through a material. Generally, unstressed amorphous polymers are considered optically isotropic, so the refractive index is equal in all directions throughout the material. However, semi-crystalline polymers or molecularly ordered polymers by mechanical or other means present differences in the refractive index along various directions. Hence, such materials are optically anisotropic and display birefringent, as in the case of the studied samples. The experimental data of refractometry under polarized light are listed in Table 4.

The refractivity measurements were conducted to determine the refractive indices along three axes: the *x* axis denotes the stretching and abrasion direction, the *y* axis denotes the direction orthogonal to imposed deformation and the *z* axis denotes the direction perpendicular to sample plane (along the film thickness). The registered values of *n_x_*, *n_y_* and *n_z_* are listed in Table 4. One may observe that prior mechanical treatment, the LDPE 0 film displays very small differences among the *n_x_*, *n_y_* and *n_z_* values. The LDPE 0-R film presents small increase of the *n_x_*, *n_y_* and *n_z_* in regard to pristine sample due to the alignment of the macromolecular chains from the superficial layers, as a result of abrasion of the surface. The sample at largest drawing degree that was not rubbed, LDPE 4-NR, presents more significant modification of the *n_x_*, *n_y_* and *n_z_* values owing to the bulk orientation of the polyethylene chains produced during stretching. However, the refractive indices of LDPE 4-NR are slightly lower than those of its counterpart that was rubbed (LDPE 4 film) since the latter displays supplementary molecular orientation generated by abrasion. All stretched and rubbed films exhibit higher differences among *n_x_*, *n_y_* and *n_z_* values, which become more pronounced as the stretching level ranges from 1.5 to 4.0. Irrespectively to applied treatment, these three parameters are decreasing as the wavelength is higher. Especially for the processed foils, the refractive indices obey the following inequality of *n_x_* > *n_z_* > *n_y_* at all wavelengths.

Moreover, the angle between the optical axes of the samples is determined. If the main system of coordinates for the studied samples is noted *xOyz* and the refractive indices measured using linearly polarized light with electric field intensity parallel to its axes are $n_{x}$, $n_{y}$ and $n_{y}$, respectively, the ellipsoid and the equation of refractive indices can be written as in Equations (8) and (9):

(8) $$\frac{\alpha_{p}^{2}}{n_{x}^{2}}+\frac{\beta_{p}^{2}}{n_{y}^{2}}+\frac{\gamma_{p}^{2}}{n_{z}^{2}}=\frac{1}{n^{2}}\;,$$

(9) $$\alpha^{2}\left(n^{2}-n_{y}^{2}\right)\left(n^{2}-n_{z}^{2}\right)+\beta^{2}\left(n^{2}-n_{x}^{2}\right)\left(n^{2}-n_{z}^{2}\right)+\gamma^{2}\left(n^{2}-n_{y}^{2}\right)\left(n^{2}-n_{x}^{2}\right),$$

In relations (8) and (9) the light propagation is parallel to versor $\vec{N}(\alpha ,\beta ,\gamma )$ and the versor of polarization is $\vec{p_{0\;}}(\alpha_{p},\beta_{p},\;\gamma_{p})$. When light propagates on *Oz* axis, the electric field of light parallel to *Oz* is ordinary component and the refractive index of the polyethylene is $n_{1}=n_{z}$. Equation (9) has two solutions: $n_{2}=n_{x\;}$(for α=0) and $n_{2}=n_{y\;}$(for α=1). The solutions of Equation (9) are one sphere of radius $n_{z}$ and an ellipsoid with semiaxes$\;n_{x\;}$, $n_{y\;}$and $n_{z}$ given by the second group of solutions. The intersections with the main plane *xOy* are one circle and one ellipse, and the optical axes of the studied samples are the intersection of the two geometrical bodies. The angle between the *Ox* and *Oy* axes is given in Table 5 for LDPE 0–LDPE 4 films.

The linear birefringence was determined along the three planes, namely: Δ*n_xy_* was evaluated along *xy* plane, Δ*n_xz_* was evaluated along *xz* plane and Δ*n_zy_* was evaluated along *zy* plane according to Equations (10)–(12):

(10) $$\Delta n_{xy}=n_{x}-n_{y}\;,$$

(11) $$\Delta n_{xz}=n_{x}-n_{z\;},$$

(12) $$\Delta n_{zy}=n_{z}-n_{y\;},$$

**Figure 9:**
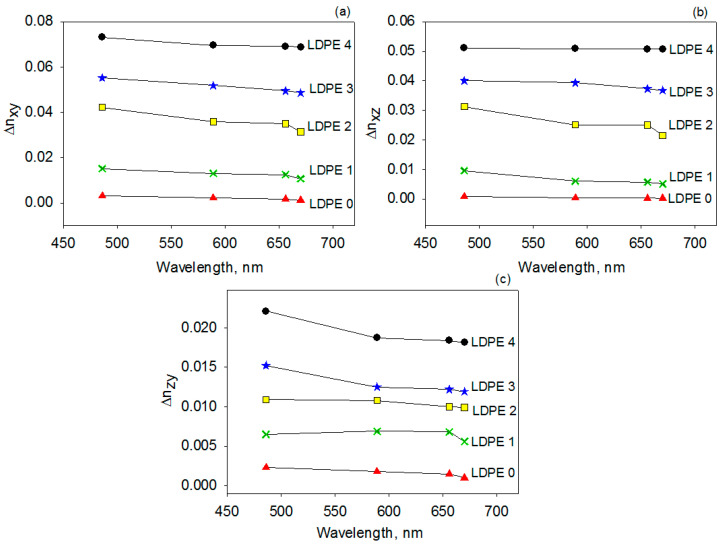
Birefringence dispersion for untreated and mechanically treated LDPE samples along the *xy* plane (**a**), *xz* plane (**b**) and *zy* plane (**c**).

The spectral dependence of the Δ*n_xy_,* Δ*n_xz_* and Δ*n_yz_* at 486 nm, 589 nm, 656 nm and 670 nm is depicted in Figure 9.

All prepared LDPE films present positive values of the linear birefringence along the three considered planes and they vary in the following order: Δ*n_xy_* > Δ*n_xz_* > Δ*n_zy_*. The magnitude of the birefringence is biggest in the *xy* plane, where both of the optical axes are placed (the drawing and abrasion of the polymer films is performed along *x* axis). The birefringence along the *xz* plane is slightly smaller comparatively to that of the *xy* plane, but higher than for the *zy* plane, which might be a result of increased surface roughness induced by combined stretching and rubbing. Furthermore, it is worth noting that the birefringence slightly changes when the light wavelength is shifting from the blue to the red part of the spectrum. On the other hand, at a constant wavelength, one may remark a gradual increase in the *Δn_xy_, Δn_xz_* and *Δn_yz_* values as the drawing degree is larger for the case of the mechanically processed samples.

#### 3.1.4. Optical Retardation

Knowing that a material with strong birefringence is needed for optical retardation uses, the mechanically processed sample characterized by the highest molecular ordering (LDPE 4) was selected for this analysis. It is known that the compensation ability of a birefringent material is affected by the thickness and the differences in refractivity along the three axes [23]. The in-plane birefringence and out-of-plane birefringence are defined by Equations (13) and (14) [23]:

(13) $$Re=\left(n_{x}-n_{y}\right)t,$$

(14) $$Rth=[\frac{\left(n_{x}+n_{y}\right)}{2}-n_{z}]t,$$

where $n_{x}$ is the refractive index along *x* axis (direction of the biggest refractive index in the plane of the sample), $n_{y}$ is the refractive indices the *y* direction (orthogonal to *x* axis), $n_{z}$ is the refractive index along the normal direction of sample surface and *t* denotes the thickness of the sample.

The out-of-plane and in-plane birefringence values enable the calculation of another key parameter for ORF applications, namely the *N_z_* coefficient, which is defined by Equation (15) [23]:

(15) $$N_{z}=\frac{n_{x}-n_{z}}{n_{x}-n_{y}},$$

**Figure 10:**
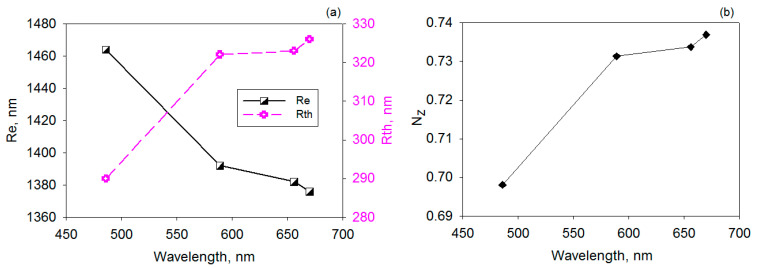
Variation of *Re* and *Rth* parameters (**a**) and *N_z_* coefficient (**b**) with wavelength for LDPE 4 film.

The dispersion data of *Re* and *Rth* parameters for LDPE 4 sample are illustrated in Figure 10a. One may notice that in-plane retardation tends to decrease upon increasing the light wavelength, while the out-of-plane retardation ranges in the opposite manner. The values of the *N_z_* coefficient increase towards higher wavelengths and presents sub-unitary values as in the case of biaxial *z* plates. This kind of optical compensation films are useful for reduction of radiation leakage in the LCD or OLED devices.

### 3.2. Analyses of LDPE Films for BPF Applications

The LDPE foils were treated by combined plasma exposure and immersion in a dye for tuning the optical absorption and surface topography to correspond for BPF uses.

#### 3.2.1. Morphology Investigation

**Figure 11:**
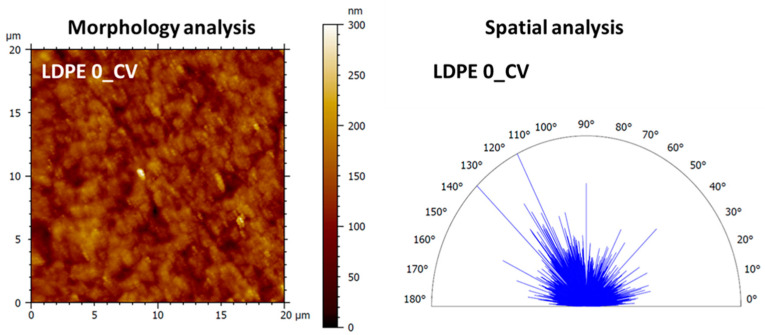
2D topographical image and corresponding polar graph of the texture direction obtained for polymer film containing the dye (LDPE 0_CV) used in morphology and spatial analyses.

The morphological analysis (Figure 11) highlighted the capacity of plasma activated pristine LDPE polymer film to adsorb and trap the CV molecules due to its high fluid retention capacity in the core and valley regions of the surface relief, reflected by the values of core void volume and valley void volume presented in Table 3. Therefore, the incorporation of the dye onto the surface of pristine polymer film results in a reduction in roughness from 38.1 nm (LDPE 0) to 27.4 nm (LDPE 0_CV) due to the light filling of relief forms. This phenomenon leads to a decrease in the complexity of the morphological formations, the surface area ratio reaching up to 1.302%. Regarding the spatial analysis, the texture direction index of the surface of 0.626 calculated from the polar graphs of the texture direction (Figure 11) and the texture isotropy of 76.85% calculated from the autocorrelation function demonstrates a pronounced isotropic nature of the surface from a morphological perspective at the micro/nanoscale.

#### 3.2.2. Colorimetry Investigation

**Figure 12:**
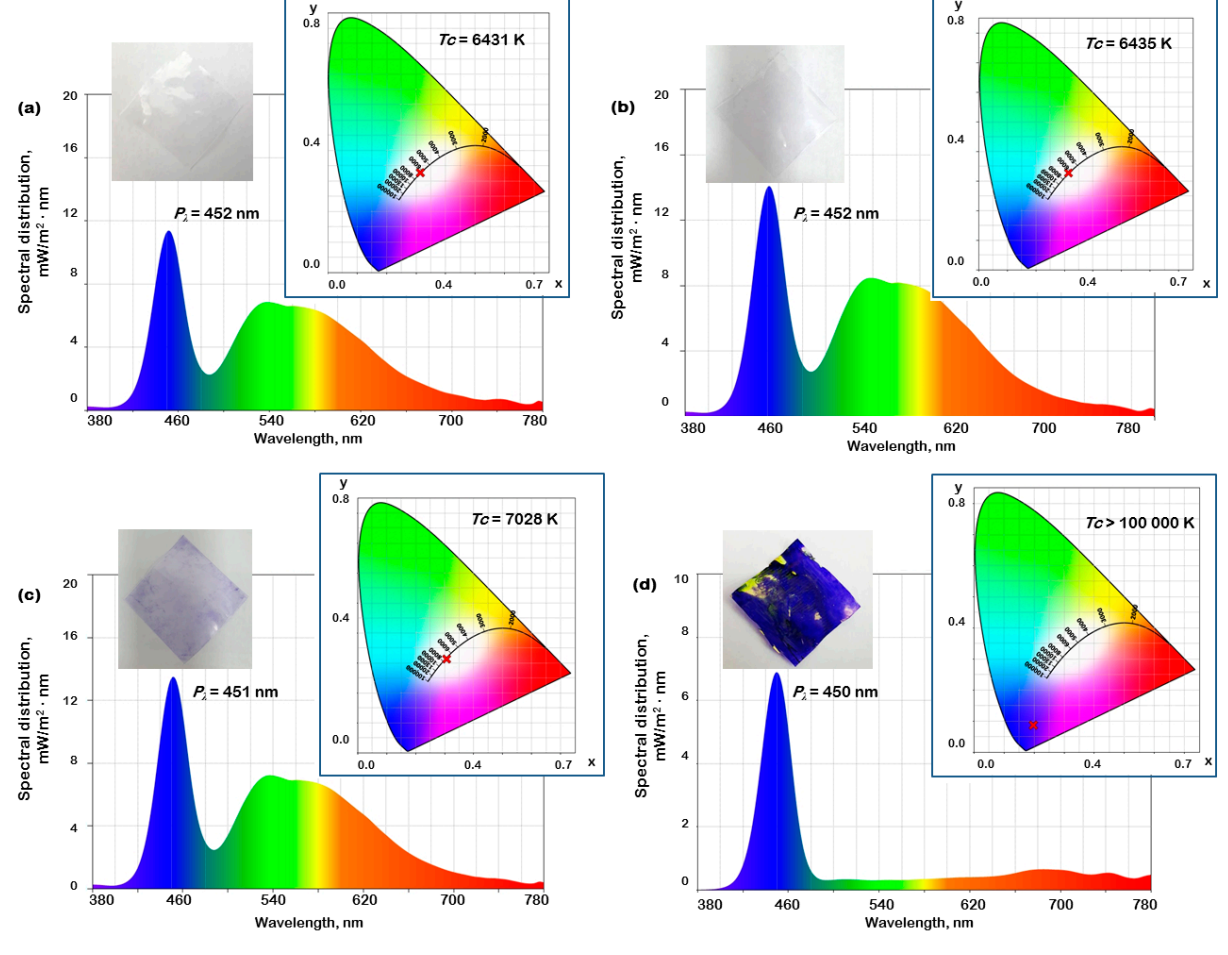
The spectral distribution, film pictures and chromaticity diagrams (CIE 1931) for LDPE 0 film (**a**), LDPE 0-P film (**b**), LDPE 0-NON-P_CV (**c**) and LDPE 0_CV film (**d**). The red cross symbol denotes the value of the *Tc*.

The colorimetric tests were conducted by placing the pristine (LDPE 0) and dyed polymer (LDPE 0_CV) layers onto the illuminance meter. For comparative reasons, the investigation also included plasma treated LDPE 0 film without CV (LDPE 0-P) and LDPE 0 film immersed in CV in the absence of plasma action (LDPE 0-NON-P_CV). The spectral distribution, which depicts the radiant exitance was recorded for all four samples. As shown in Figure 12a, the pristine LDPE 0 foil presents a maximum peak wavelength (*P_λ_*) at 452 nm and a broader band over 450–700 nm range, which is a typical white color spectrum. This is consistent with the transparent aspect of the sample shown in the inset picture of the LDPE 0. As noted in Figure 12b, the LDPE 0-P sample presents similar spectral distribution characteristics to pristine polyethylene foil, while LDPE 0-NON-P_CV only differs in terms of maximum peak wavelength, which is slightly lowered to 451 nm (Figure 12c). Plasma exposure is known to generate higher surface roughness and larger wettability, which are useful for the incorporation of the dye molecules onto the studied material’s surface. This can be observed in the picture image of the LDPE 0_CV foil, presented in inset of Figure 12d, which reveals a blue-violet color. Such an aspect is reflected on the radiant exitance of LDPE 0_CV foil, which presents only a sharp peak at 450 nm (see Figure 12d).

The color temperature (*Tc*), which denotes the level of ‘warmth’ (yellow) or ‘coolness’ (blue) of the radiation emitted by a source, is measured for all four samples. The recorded data are included in the chromaticity diagrams (CIE 1931) that are illustrated in Figure 12a–d. The LDPE 0 film presents the lowest value of the *Tc* of 6431 K, which corresponds to a warmer spectrum. Plasma treatment produces a slight increase of the *Tc* up to 6435 K for LDPE 0-P. The presence of the CV dye generates considerable enhancement of the *Tc* up to 7028 K for LDPE 0-NON-P_CV and even more when the dye is bonded via plasma irradiation, namely > 100,000 K for LDPE 0_CV. Higher values of the temperature color observed for the dyed polymer samples (LDPE 0-NON-P_CV and LDPE 0_CV) can be attributed to the blue or cooler colors from the spectrum. However, the differences between the *Tc* of these two dyed polyethylene foils indicate that plasma has a significant role on the dye attachment to the sample surface (as supported by the pictures of the samples from Figure 12c,d).

The CIE color spaces are strongly influenced by the presence of the triphenylmethane derivative. The normalized values of tristimulus parameters (*X*_0_, *Y*_0_ and *Z*_0_) facilitate the calculation of the CIELAB coordinates (L*, a* and b*), according to Equations (16)–(18) [67]:

L* = 116(*Y*_0_/*Y_n_*)^1/3^ − 16, (16)

a* = 500[(*X*_0_/*X_n_*)^1/3^ − (*Y*_0_/*Y_n_*)^1/3^], (17)

b* = 200[(*Y*_0_/*Y_n_*)^1/3^ − (*Z*_0_/*Z_n_*)^1/3^], (18)

where L* is the lightness parameter (0–black, 100–white), a* is the red/green factor and b* is the yellow/blue factor, whilst *X_n_*, *Y_n_*, *Z_n_* refer to the reference tristimulus data.

**Figure 13:**
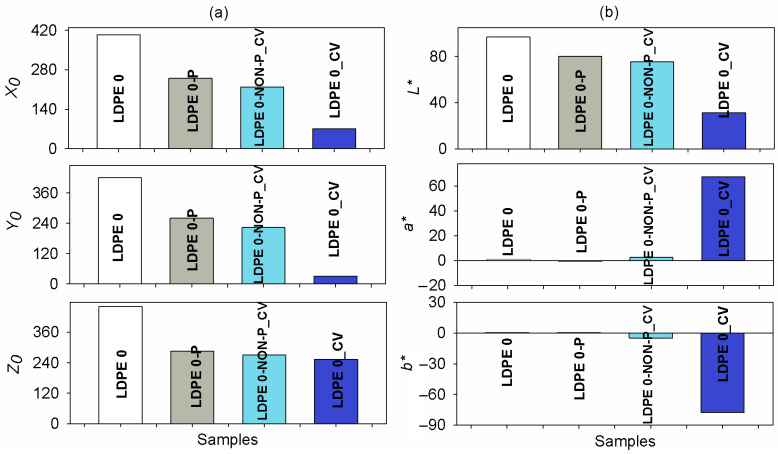
The variation of the tristimulus (**a**) and CIELAB parameters (L*, a*, b*) (**b**) for the studied LDPE 0, LDPE 0-P, LDPE 0-NON-P_CV and LDPE 0_CV films.

Figure 13a shows the measured data for the *X*_0_, *Y*_0_ and *Z*_0_ parameters and also includes the calculated values of the CIELAB parameters (L*, a* and b*) for the studied samples. As noted in Figure 13b, the lightness factor L* slightly decreases after plasma irradiation, namely from 96.8 for LDPE 0 film to 80.1 for LDPE 0-P one. Significant changes of L* are noted when the CV is present in the samples namely 75.3 for LDPE 0-NON-P_CV foil and 31.2 for LDPE 0_CV one. This denotes a diminishment of the lightness caused by the dye, especially for the plasma-irradiated and dye-treated polyethylene film.

More relevant aspects can be extracted from the analysis of a* and b* colorimetric properties. The initial LDPE 0 foil presents a small (near 0) and positive value of a* (0.05), as a result of the neutral hue of this sample. Plasma exposure determines a small modification of a* parameter which becomes negative (−0.01) for LDPE 0-P, which could be interpreted as a tiny change in hue towards a green color. In the absence of plasma action, the polyethylene foil immersed in CV solution acquires a positive and slightly higher values of a* (2.6 for LDPE 0-NON-P_CV). The LDPE 0_CV film exhibits a sharp increase of a* (67.42), which denotes the predominance of a red hue. Moreover, the positive value of b* corresponds to the pale yellow hue of the LDPE 0 (0.47) and LDPE 0-P (0.41) films. The procedure of polyethylene dyeing leads to dropping towards negative values of b* parameter, namely −5.1 for the LDPE 0-NON-P_CV and −77.58 for the LDPE 0_CV films. The change of the b* is a strongly indicative of the prevalence of a blue hue, especially for LDPE 0_CV, as a result of beneficial role of the plasma exposure prior to the dyeing of the sample (LDPE 0_CV). This confirms that the dye is better incorporated in the LDPE 0_CV foil comparatively to the LDPE 0-NON-P_CV specimen.

#### 3.2.3. UV-VIS Spectroscopy Investigation

**Figure 14:**
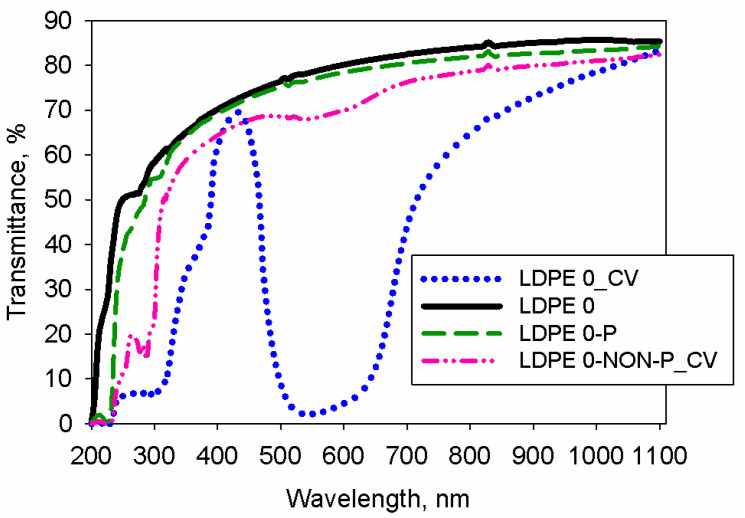
UV-VIS spectra of LDPE 0, LDPE 0-P, LDPE 0-NON-P_CV and LDPE 0_CV films.

As previously shown in this paper, the LDPE 0 film presents high transmittance over the visible domain (see the continuous black line from Figure 14). Plasma exposure of this polymer increases the surface roughness and this determines slight light scattering, and thus the transmittance of LDPE 0-P decreases to a very low extent (see the dash green curve from Figure 14). The selected dye is known to display specific spectral features, so its UV-VIS spectrum contains a main absorption band at about 580 nm and two small ones in UV zone (under 390 nm) [68]. The polyethylene foil which was treated with CV (no plasma) has a similar shape of its UV-VIS spectrum to that recorded for LDPE 0. Hence, in the case of LDPE 0-NON-P_CV, the spectrum presents a lower level of transmittance and a small shoulder around 484–643 nm. The latter might be result of low absorption caused by the few CV molecules adsorbed to the polymer surface (see the dash-dot-dot pink curve from Figure 14). The treatment by plasma exposure and immersion in the solution of the triphenylmethane derivative determines the dye attachment onto the polyethylene; hence, the spectral features of LDPE 0_CV are drastically changed (see the dotted blue curve from Figure 14). According to Figure 14, the specimen LDPE 0_CV presents a different shape of the transmission versus wavelength plot in comparison to that of the pristine polyethylene film. Thus, the spectral data of the dyed polymer reflect the electronic excitations within the triphenylmethane derivative, which are rendering the absorption bands. One may observe that LDPE 0_CV exhibits a sudden drop in the transmission values in the intervals of 200–300 nm and 480–660 nm, while other wavelengths are passing through the sample.

To acquire a proper image of the properties of the studied BPF, a comparison of the key performance parameters of LDPE 0_CV and other polymer/dye systems from literature [49,69,70] is undertaken. As seen in Table 6, several parameters were analyzed, namely: center wavelength (CW), peak transmission (PT), full width at half maximum (FWHM) and wavelengths corresponding to absorption band (WAB). One may observe that the values of CV and PT are dependent on the structure of the used polymer (LDPE, PMMA or polyvinyl alcohol (PVA)). Additionally, the FWHM of LDPE 0_CV is under that of PVA_CV and PMMA_CV, but it is larger than that reported for PMMA_BCB. Moreover, the investigated sample blocks radiation on a wider range than the PVA_CV, but on a smaller domain comparatively to PMMA dyed with either CV or brilliant cresyl blue (BCB). The observed spectral features of LDPE 0_CV are recommending this material as being a good candidate for BPF uses.

## 4. Conclusions

This work explores the implication on the optical and morphological properties of LDPE which were adapted by two kinds of treatments with the purpose of making ORFs or BPFs for display technologies.

Related to ORFs, there are new aspects related to the original mechanical treatment of the LDPE films, investigating its implications on the generated optical and morphological anisotropy. Dichroic ATR-FTIR spectroscopy results show relevant spectral changes were induced in polyethylene by stretching and combined stretching and abrasion. Polarized light microscopy images reveal the appearance of striations following the direction of mechanical stretching which overlap with the grooves produced upon abrasion of the samples (in the same direction). AFM analyses revealed an increase in surface roughness after the mechanical processing of the polymer film. In addition, the proposed treatment determines the molecular orientation that is reflected at the surface morphology level through a decrease in the texture direction index of the surface. UV-VIS analyses show that the samples are transparent and, after their mechanical modification, the specimens have different transparency along and orthogonal to the direction of external forces. To evaluate the level of molecular orientation generated in the samples, birefringence measurements were performed. This parameter decreases slightly with wavelength and increases as the level of stretching is larger. The in-plane optical retardation decreases with wavelength, while the out-of-plane optical retardation varies in the opposite way. The *Nz* coefficient has values lower than 1, which indicates that the LDPE films have typical anisotropy to biaxial compensation films, particularly z-plate type. Among them, the LDPE 4 film has a strong birefringence, which allows introducing a phase shift among the distinct polarization components of incoming light, thus adjusting the polarization state prior passing via the nematic layer. This is beneficial for finer control over radiation transmission and implicitly on the image contrast shown on the screen, enhancing its quality.

Concerning BPFs, the novel treatment of plasma exposure and immersion in triphenylmethane derivative led to significant modification of surface morphology, the sample presenting a decrease in the complexity of the morphological formations. Colorimetric parameters support the change towards blue hues as a result of dye attachment to the polyethylene foil. UV-VIS spectra reveal that this material blocks radiation in 200–300 nm and 480–660 nm, while leaving other wavelengths to pass, as demanded by the pursued application. Such optical components are inserted into the backlight unit of a display to improve the backlight spectrum. The obtained BPF transmits blue-green and red colors; therefore, the LDPE 0_CV film has the potential to enhance the color gamut of LCD devices.

## Figures and Tables

**Table 1:** The sample acronyms, thickness, width, length and *Rs* ratio between final length (upon stretching) and initial one (no stretching).

Sample Acronym	Thickness, µm	Width, cm	Length, cm	*Rs*
LDPE 0	70	1.1	6.4	1.0
LDPE 1	60	0.9	10.0	1.6
LDPE 2	40	0.7	15.3	2.4
LDPE 3	30	0.6	20.1	3.1
LDPE 4	20	0.4	25.6	4.0

**Table 2:** The 3D texture parameters characteristic for the morphology and furrows analyses.

3D AFM Texture Parameters	Samples			
	LDPE 0	LDPE 0-R	LDPE 4-NR	LDPE 4
***Morphology analysis***				
Root mean square roughness, *Sq* (nm)	38.1	55.0	90.9	141.2
Surface area ratio, *Sdr* (%)	2.463	2.500	2.755	7.615
***Furrows analysis***				
Maximum depth of furrows	164.4	195.2	285.2	482.4
Mean depth of furrows	56.8	84.26	122.4	217.8

**Table 3:** The 3D texture parameters characteristic for the functional and spatial analyses.

3D AFM Texture Parameters	Samples			
	LDPE 0	LDPE 0-R	LDPE 4-NR	LDPE 4
***Functional analysis***				
Peak material volume, *Vmp* (nm^3^/nm^2^)	1.78	2.00	5.74	4.09
Core material volume, *Vmc* (nm^3^/nm^2^)	34.03	47.77	100.80	134.80
Core void volume, *Vvc* (nm^3^/nm^2^)	44.00	54.40	88.67	184.70
Valley void volume, *Vvv* (nm^3^/nm^2^)	4.66	8.8	11.59	13.87
***Spatial analysis***				
Texture direction index of the surface, *Stdi*	0.626	0.242	0.246	0.167

**Table 4:** The values of the *n_x_*, *n_y_* and *n_z_* measured at different wavelengths for the pristine and mechanically treated LDPE films.

Sample	Refractive Index	Wavelength, nm			
		486	589	656	670
LDPE 0	*n_x_*	1.5204	1.5182	1.5170	1.5131
	*n_y_*	1.5173	1.5160	1.5152	1.5119
	*n_z_*	1.5196	1.5178	1.5167	1.5129
LDPE 0-R	*n_x_*	1.5266	1.5251	1.5237	1.5129
	*n_y_*	1.5230	1.5226	1.5216	1.5111
	*n_z_*	1.5257	1.5242	1.5224	1.5126
LDPE 1	*n_x_*	1.5275	1.5225	1.5192	1.5162
	*n_y_*	1.5124	1.5095	1.5067	1.5055
	*n_z_*	1.5179	1.5164	1.5135	1.5111
LDPE 2	*n_x_*	1.5489	1.5349	1.5328	1.5232
	*n_y_*	1.5067	1.4990	1.4978	1.4918
	*n_z_*	1.5176	1.5098	1.5078	1.5017
LDPE 3	*n_x_*	1.5568	1.5442	1.5409	1.5337
	*n_y_*	1.5016	1.4924	1.4914	1.4851
	*n_z_*	1.5168	1.5049	1.5036	1.4970
LDPE 4-NR	*n_x_*	1.5624	1.5492	1.5480	1.5457
	*n_y_*	1.4976	1.4855	1.4851	1.4836
	*n_z_*	1.5117	1.4985	1.4958	1.4945
LDPE 4	*n_x_*	1.5673	1.5554	1.5540	1.5471
	*n_y_*	1.4941	1.4858	1.4849	1.4783
	*n_z_*	1.5162	1.5045	1.5033	1.4964

**Table 5:** Angle between the optical axes of LDPE sample.

Sample	Angle, °			
	486 nm	589 nm	656 nm	670 nm
LDPE 0	60.9847	50.4307	48.1517	48.1824
LDPE 1	105.3437	86.1041	84.5978	87.0176
LDPE 2	117.8654	112.5384	114.4775	123.2490
LDPE 3	114.9909	119.8952	119.2537	119.4861
LDPE 4	111.4419	115.8032	116.1297	116.5516

**Table 6:** The values of CW, PT, FWHM and WAB of the studied LDPE 0_CV film and other polymer/dye systems.

System	CW, nm	PT, %	FWHM, nm	WAB, nm	Reference
LDPE 0_CV	429	70.2	349–480	200–300 480–660	This work
PVA_CV	413	88.3	316–496	243–259 294–312 506–633	[49]
PMMA_CV	424	81.0	up to 510	531–622	[69]
PMMA_BCB	478	46.1	455–532	200–400 538–669	[70]

## Data Availability

Dataset available on request from the authors.
